# Ultrasound imaging versus morphopathology in cardiovascular diseases. Coronary collateral circulation and atherosclerotic plaque

**DOI:** 10.1186/1476-7120-3-6

**Published:** 2005-03-01

**Authors:** Giorgio Baroldi, Riccardo Bigi, Lauro Cortigiani

**Affiliations:** 1 Institute of Clinical Physiology, National Research Council, Milan and Pisa, Italy; 2 University School of Medicine and "A. De Gasperis" Foundation, Niguarda Hospital, Milan, Italy; 3 Cardiovascular Unit, "Campo di Marte" Hospital, Lucca, Italy

## Abstract

This review article is aimed at comparing the results of histopathological and clinical imaging studies to assess coronary collateral circulation in humans. The role of collaterals, as emerging from morphological studies in both normal and atherosclerotic coronary vessels, is described; in addition, present role and future perpectives of echocardiographic techniques in assessing collateral circulation are briefly summarized.

## 

In the past 25 years, the concept of a compensatory function of the coronary collaterals (or anastomoses) – i.e. vessels that join different coronary arteries or branches – has been practically cancelled from the mind of cardiologists since cineangiography shows that the onset of coronary heart disease (CHD) occurs independently of their presence. The assumption, therefore, was and is that they have no compensatory meaning [1] and coronary obstruction causes ischemia. A crucial and questionable assumption which disregards solid and recognized pathological data and supports invasive therapies, the diagnostic gold standard being the coronary cineangiography. In many cardiological centers, at the first chest discomfort, the latter is the guide for emergency angioplasty + stent or surgical bypass when a coronary ostruction is found; with the belief that a severe coronary stenosis causes angina pectoris, its occlusion an acute myocardial infarct (AMI) or sudden death (SD) and chronic ischemia explains hibernating myocardium.

By injection under controlled pressure of plastic materials through the aorta, casts of coronary arteries, including coronary ostia, in normal and pathological hearts were obtained. They gave an objective tridimensional view of anatomy, different patterns of coronary distribution and overall collaterals in relation to coronary lumen reduction. The method allowed a histologic control of the myocardium [2-4]. The casts of normal coronary arteries showed a smooth surface without identations easily identified when even a minor lumen reduction was present. In hearts of normal people dead by accident without pathological findings at autopsy, *homocoronary *(between branches of the same coronary artery) and *intercoronary *(between different coronary arteries) anastomoses were present everywhere joining at any level the intramural branches. Only in two of more than 600 hearts, superficial collaterals between extramural coronary arteries were seen and sampled for histology. The diameter of the innumerable normal collaterals ranged from 20 (maximal penetration of plastic injection) to 350 microns, frequently assuming a corkscrew aspect, possible adaptation to the contraction cycle of the myocardium (Figure 1). The first conclusion was that arterial intramural system, including the terminal bed, is an anastomotic network, at least from the anatomical viewpoint.

**Figure 1:**
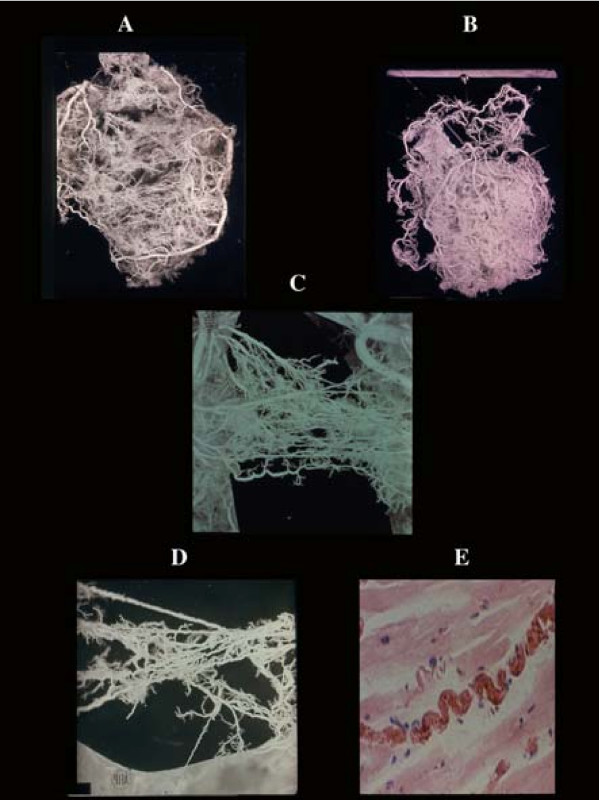
Coronary anastomoses or collaterals. A) intercoronary ventricular and (B), atrial. C) homocoronary anastomoses. Note the innumerous collaterals joining different intramural branches at any level of their course. They have frequently a corkscrew aspect (D) visible also histologically (E), as adaptation to cardiac contraction-relaxation cycle.

In hypertrophic hearts with normal coronary arteries and in normal hearts of patients with chronic hypoxia, e.g. anemia, collateral diameter and length were increased in the whole intramural system (500 microns; Figure 2). The more impressive change was seen in presence of coronary stenosis greater than 70% with a diameter and length exceeding 1000 microns and several centimeters respectively (Figure 3). The other peculiarity was that collateral enlargement was strictly related to a stenosis filling distal tract of the obstructed vessel (*satellite anastomoses*); when more than one severe stenoses exist each one had its own satellite collaterals. However, an identical obstruction located at the same level of an artery might show relatively few highly enlarged collaterals (the only ones visible by cineangiography), or numerous relatively small collaterals (Figure 4). A finding possibly due to a redistribution of blood flow consequent to newly formed severe stenoses or an infarct. In the latter condition, all vessels within the necrotic tissue disappear (*avascular area *seen in plastic casts; Figure 5) and the surviving collaterals at periphery will further enlarge since the pressure gradient distal to the coronary obstruction persists.

**Figure 2:**
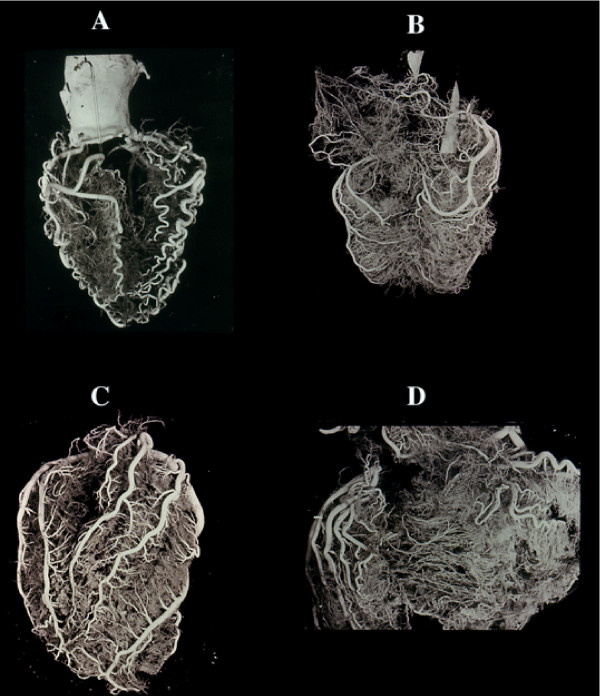
Vessel changes in relation to modification of the cardiac mass. A) atrophic heart with acquired serpentoid form of extramural vessels due to cardiac mass reduction, and minor intramural vascularity. The contrary is seen in cardiac hypertrophy (B) in which the extramural arteries increase in length and diameter (but not in number) to adapt themselves to the greater myocardial mass. Similarly, the same enlargement is seen in the intramural branches. Cor pulmonale, in which condition the right ventricle may become greater than the left one, is an extreme example of adaptation of extramural (C) and intramural, including collaterals (D). No histologic evidence exists of new vessel formation. The cardiac vein show a similar behaviour.

**Figure 3:**
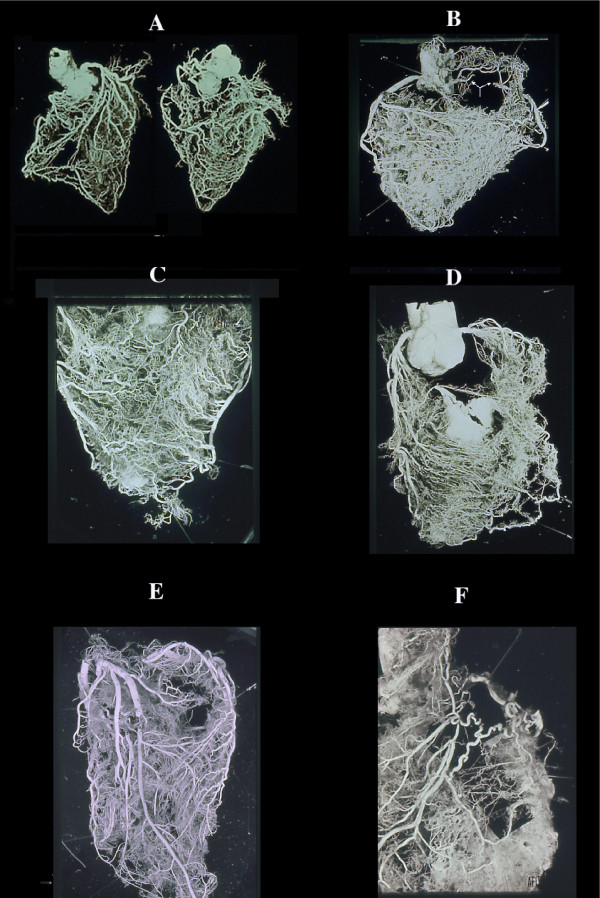
Collateral enlargement in topographical relation (satellite) with severe stenosis or occlusion. A double occlusion of LAD (anterior view) and occlusion of RCA (posterior view) apparently compensated by enlarged collaterals in a non cardiac patient dead from brain hemorrhage. B, similar condition in cases with RCA occlusion (arrow) without corresponding myocardial infarct with numerous homo and intercoronary collaterals of the anterior wall (C), and (D) septum. Occlusion of LAD without evidence of other stenotic changes of the coronary arteries in a 39-year-old woman with rheumatic heart disease and mitral insufficiency. In this case, arteritis was documented histologically by sampling before corrosion. An acute infarct (avascular area at the apex, arrow) was present. F, a single, high enlarged collateral from LCX, supplying the distal tract of an occluded LAD. Note, numerous normal anastomoses. This indicates that ischemia is not the cause (no diffuse enlargement of all collaterals in the whole ischemic area) but rather pressure gradient induces selective compensatory routes.

**Figure 4:**
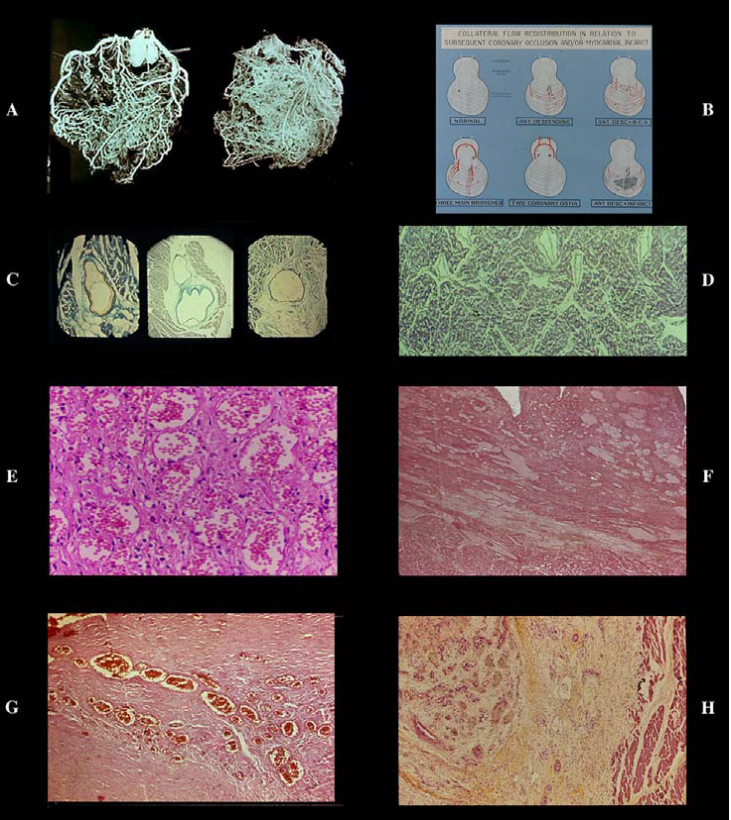
Different aspects of collateral compensation in presence of the same occlusive pattern of LAD. A, relatively few very enlarged collaterals and (B) numerous relatively small collaterals. This divergency may be due to progressive atherosclerotic obstruction of other main vessels or lost of the intramural vasculature, including collaterals, following an infarct. Chart C shows all the possibilities of flow redistribution. The histology of the enlarged anastomoses corresponds to a capillar-like wall, even in the rare extramural collaterals with rudimentary focal tunica media (C). D), enlarged collaterals in a case of anomalous origin of LAD from the pulmonary artery and (E,G) different aspects of giant capillaries (or plexus) in various stages of an acute/old infarction. The absence of new vessel formation is well documented in recent infarcts associated with endocardial thrombus (G). In the latter numerous new vessels form in the granulation tissue repair of the thrombus in contrast to their absence in infarct (arrow; postmortem coronary injection for vessels identification).

**Figure 5:**
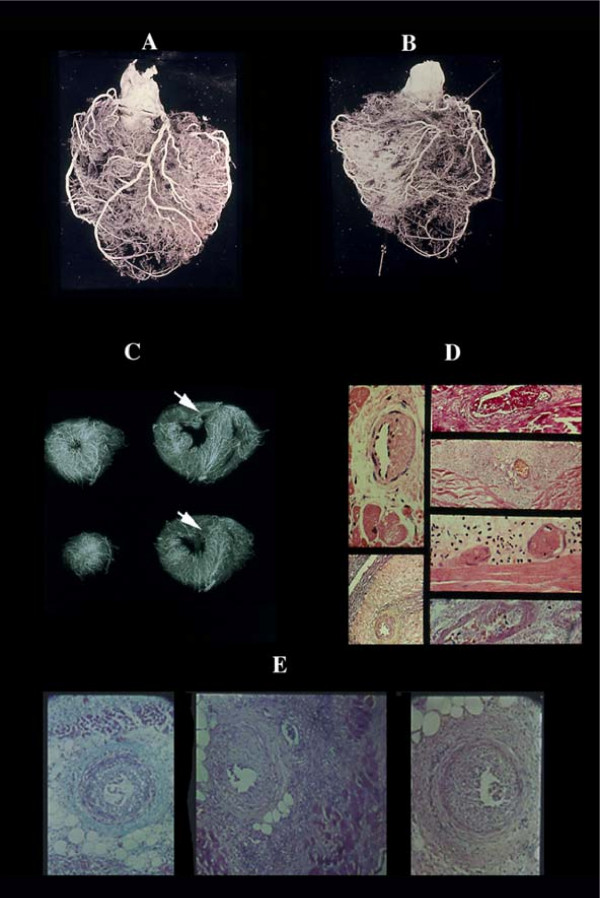
Avascular area of an infarct. By plastic cast (A anterior, B posterior view) or postmortem angiogram (C) the infarcted zone (arrow) lacks of intramural vessel injection ("avascular area"). Stretching of the necrotic myocardium and secondary vascular damage with wall degeneration and thrombosis (D), explain this vascular "sequestration" which occurs in early phase. This may indicate a blockage without possibility of therapeutical intervention via blood flow within the infarcted myocardium. Note that the avascular area in this AMI case documented histologically, depended from LAD without evidence of occlusion or severe stenosis. The occluded vessel (arrow) was (B) the RCA, the distal part of which was filled by numerous anastomoses. No myocardial damage was seen in its territory. By dissection even an expert pathologist, the diagnosis could be of myocardial infarction following occlusion of the RCA. E) obliterative intimal hyperplasia in arterioles around a seven days old infarct with early repair process.

Another satellite collateral system is annexed around and within the atheroclerotic plaque. Plastic casts and histological serial sections showed an extensive vascularization limited only at plaque level and formed by giant adventitial capillary-like vessels filled by intracoronary radiopaque injected material, connecting secondary branches proximal and distal to the stenosis as well as new vessels formed within the atherosclerotic intima i.e. arterioles, with a well developed tunica media, related to angiomatous plexuses which open in the residual lumen (Figure 6). This *plaque satellite system *may explain why by cineangiography the coronary tract distal to stenosis is immediately filled while in its absence a delay or flow reduction should be expected.

**Figure 6:**
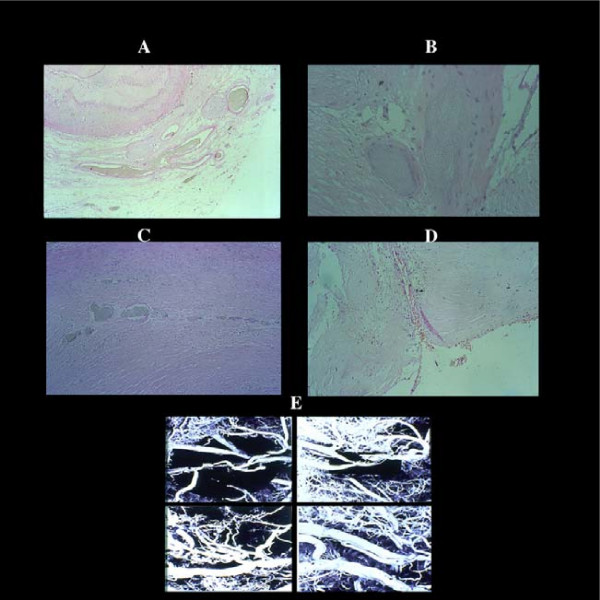
Vascularization of a coronary atherosclerotic plaque showing different aspects of neovascularization. By serial sections of postmortem injected plaques, giant advential capillary-like vessels (A) are connected with secondary branches proximal and distal to the plaque and with new arterioles (B) with a well developed tunica media (indication of functioning blood flow), within the thickened, atherosclerotic intima in turn joined through angiomatous plexuses (C) to the residual lumen (D) E) plastic casts of plaques with different aspects of vascularization.

Both homo-intercoronary and plaque collateral systems are anatomical structures capable to adapt in particular pathological conditions. The question is whether or not they are able to prevent ischemia and compensate an occlusion which by cineangiography appears as a "cut off" of a vessel without imaging of its distal tract. It must be stressed that in postmortem casts with coronary occlusion the latter was always injected through collaterals.

In 87% of AMI patients, within four hours from clinical onset, a cineangiographic occlusion was observed and in 88% of cases undergone emergency bypass surgery, a "layered thrombus" was recovered "proximal to stenosis" [5]; a thrombus due to plaque rupture [6-8] causing the infarct or sudden death.

In discussing this dogma the first need is to review the function of the collaterals.

## Collateral function

### Capillary function in presence of normal coronary arteries

In normal hearts and in pathologic hearts with normal coronary arteries, the collaterals, due to their capillary structure, participate to the metabolic exchange as terminal capillary bed. This means a much greater extent of the exchange surface which invalidates any "one myocardial / one capillary" model to study the delivery of any substance from capillary to myocardial cell. The myocardial interstitium is crossed by a myriad of "endothelial" vessels in any direction.

### Compensatory function in presence of coronary obstruction

The demonstration of tridimensional collateral enlargement by casts indicates, per se, that there was an increased blood flow. Their adequacy to compensate one or more severe coronary obstructions is documented by the following main facts:

1. At the first episode of coronary heart disease (CHD) in apparently healthy people acting their normal life, 89% with a fatal AMI had one or more (47%) severe atherosclerotic stenosis greater than 70% ;65% of sudden and unexpected death (SUD) showed the same finding in one or more (35%) vessels; 66% of non cardiac patients dead from other diseases and 39% of normal subjects dying from accident had the same severe atherosclerotic stenosis in one or more (40% and 16% respectively) coronary arteries (Table 1). At histology, all plaques were old lesions preexisting months or years without any evidence of CHD despite a stressful life and in absence of a myocardial infarct. The only explanation is that the collateral system was able to fully compensate the blood flow reduction consequent to the stenoses.

**Table 1:** Maximal atherosclerotic lumen diameter reduction and number of main arteries with severe (≥ 70%) stenosis

**Source**	**Acute myocardial infarct**		**Sudden death unexpected**		**Non cardiac atherosclerotic Patients**	**Accidental death in normal people**
	**1st**	**chronic**	**1st**	**chronic**		
**Cases**	**145**	**55**	**133**	**75**	**100**	**97**

**% Lumen reduction**						
0	3	-	10	-	7	8
<50	3	1	18	-	10	20
50–69	10	-	18	5	17	31
70–79	30	8	21	8	11	19
80–89	45	11	39	14	24	13
≥ 90	54	35	27	48	31	6
**No. arteries ≥ 70%**						
1	61	16	40	13	26	22
2	49	22	34	26	18	13
> 3	19	16	13	31	22	3

1^st ^episode, in apparently normal people without extensive monofocal myocardial fibrosis Chronic, in subjects with history of coronary heart disease and/or extensive myofibrosis.

2. Myocardial infarct size measured planimetrically was not related to the number of severe coronary stenoses found in each AMI case (Table 2) as should be. More severe coronary stenoses should determine a higher ischemia resulting in larger infarcts.

**Table 2:** Lack of correlation between number of severe (≥ 70%) coronary stenoses and acute infarct size (% left ventricular mass) in 200 consecutive and selected fatal cases.

**Source**	**Acute myocardial infarct**		
**Cases**		**200**	
	**97**		**103**
	**≤ 20**	**size**	**> 20**
	
Lumen reduction			
< 69	7		10
≥ 70	90		93
in 1	39		38
2	37		34
≥ 3 vessels	14		21

p < 0.05 for trend

3. No relation between the total vascular territory of obstructed coronary artery and infarct size which often extended in territories of non stenosed or occluded vessels. In vivo hypokinetic zones expand in well perfused region [9].

4. The relatively frequent finding of a coronary occlusion without an infarct.

5. In an experiment done in a leading dog lab, a controlled coronary stenosis, maintained for few days and then occluded, did not determine any dysfunction or infarct because a dramatic increase of collateral flow [10-12].

These are the main facts supporting the concept that collaterals shown postmortem succeed in limiting or abolishing ischemia induced by coronary obstruction and question the existence of chronic ischemia due to coronary atherosclerosis since a plaque takes time to develop while collaterals [10,11] adapt itself quickly as soon a pressure gradient between stenosis and distal vessel is established. On the other hand, there is no demonstration of a possible failure, both acute or chronic, of collaterals; including spasm since they have not tunica media.

The inability of cineangio imaging to visualize collateral systems is explained by its very limited power of resolution of all intramural vessels and by the selective injection of radiopaque labelled blood flow in one coronary artery competing with non labelled flow from the other coronary artery. Only very enlarged intercoronary anastomoses can be seen cineangiographically without any value in relation to cardiac dysfunction. Acute ischemia induced by balloon inflation at angioplasty may depend on sudden occlusion by compression of the collateral plaque system.

### Active coronary atherosclerotic plaque according to cineangio imaging

Active plaque means an impending infarct expressed by a variety of angiographic signs as irregular lumen, haziness with ill-defined margins, smudge appearance, inhomogeneity, opacification, luciencies, persistence of radiopaque material, etc. Signs difficult to correlate with postmortem findings since terminal changes can not be excluded. They may represent the irregular vascularization of the atherosclerotic plaque opacified by the injected radiopaque material. Worthy of note is that cineangio defects can persist unchanged per years [13].

### Cineangio coronary occlusion

The very high frequency of coronary occlusion seen angiographically in AMI patients (see above) does not correspond to that observed in pathological studies in which the mean figure is 50% for AMI and 29% for SUD patients. Nevertheless, different selection of material, divergent definition and an absence of a correct correlation of all pertinent variables give reason of dissimilar conclusions. In 200 selected AMIs and 208 SUD cases the unique cause of occlusion was a thrombus found in 41% and 29% respectively. In AMI group it correlated significantly with a lumen reduction greater than 70% (93%), length of plaque more than 6 millimeters (95%), its concentric shape (100%), prevailing atheroma (84%), medial neuritis (92%) infarct size greater than 50% (86%). SUD cases showed a similar behavior.

**Figure 7:**
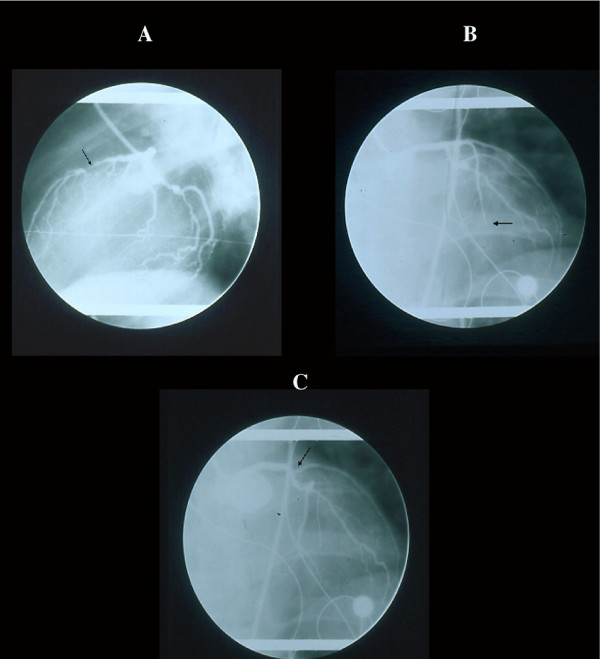
Cineangiographic monitoring in a patient with non occlusive LAD stenosis (A) who developed an extensive infarct without angiographic occlusion. The subsequent imaging of occlusion began distally (B) and ascended to the origin (C) of the vessel (arrow) indicating that the angiographic "pseudocclusion" was due to stasis for increased peripheral resistance and not for primitive thrombosis, not shown morphologically (see text).

In reality, both clinicians and pathologists observe a phenomenon which started before, missing its onset and sequence of events to distinguish whether primary or secondary. In only one case reported in literature [14], this sequence and histological examination of the whole heart was possible in a 45 year old man suffering a two months unstable angina. At coronary cineangiography there were two critical stenoses of the left anterior descending branch (LAD), one proximal and another distal to the origin of diagonal branch and a critical stenosis in the first tract of the right coronary artery (RCA). An antero-septal-lateral hypokinesis was documented. After the fourth left coronary injection, in absence of any symptom or sign and cineangio imaging changes, a first ECG showed downsloped ST segment. The latter persisted for other four LAD injections when the vessel disappeared, again, without any subjective and objective signal. Intracoronary vasodilator and fibrinolytic agents, successful angioplasty in reopening critical stenoses, surgical bypass in rapid sequence were performed without re-establishing flow. Only for few short periods a reflow occurred with an imaging of occlusion which from the distal tract ascended till the origin of LAD (Fig. 7) and not at the site of angioplastically reopened stenoses. An interesting note is that a severe chest pain started after angioplasty, 90 minutes from the first ECG change. The patient survived an extensive myocardial infarction and 12 months later underwent heart transplantation because irreversible congestive heart failure. We had the opportunity to examine the heart removed at surgery confirming a large (40% of the total left ventricular mass) antero-septal-lateral scar, end result of the infarct, scattered foci of fibrosis everywhere, absence of small vessel disease, colliquative myocytolysis expression of congestive failure, severe lumen reduction by sclerosis of LAD (90%) – despite it showed a normal lumen at bypass surgery – and vein graft (80%) without evidence of thrombosis, RCA occlusion by an organized thrombus located in an atherosclerotic plaque with 90% lumen reduction, medial neuritis i.e. lympho-plasmacellular inflammation of nerves closed to the tunica media, in all atherosclerotic plaques, absence of an infarct in RCA territory.

One case is only one case but when for the first time shows how the events developed, it becomes a precious mile stone for our knowledge demonstrating that the cineangio occlusion was a pseudocclusion namely a blood flow stasis in LAD secondary to an increased intramyocardial resistance. The first main question is how many of the 87% cineangio occlusion are pseudocclusion and whether the "layered" thrombus recovered at bypass surgery was a true thrombus or a coagulum which frequently show a layering of blood elements not seen in thrombus formation.

"Red" thrombus, namely a coagulum, is frequently and erroneously considered as thrombus. The second question concerns the nature of increased intramyocardial resistance: spasm of intramural arterial vessels or their extravascular compression by an asynergic myocardium? The first sign of CHD is hypokinesis of a myocardial zone which particularly in systole may compress vessels. Any time there is an increase of the intraventricular pressure with bulging of hypokinetic myocardium such a compression may abolish blood flow with subsequent infarction. In the reported patient location and infarct size corresponded to the hypokinetic area observed before the infarct onset.

A last comment deserves the supposition that small atherosclerotic plaques undetectable at cineangio, may rupture causing an infarct. A supposition based on the cineangio finding of a non critical stenosis observed in a vessel tributary of a territory in which an infarct will develop. Since, when the latter occurred, stenoses in other non supplying vessels did not show a further lumen reduction, the conclusion was that even the plaque related to infarction had a non critical lumen reduction [15]. A conclusion that ignores the following two main facts. First that no one pathological study demonstrated the rupture of a small plaque associated with a thrombus occluding a normal or mild stenotic lumen. Second, myocardial asynergy by increasing intramyocardial resistance, promotes plaque progression by an increased dynamic stress on wall of the supplying extramural artery. For instance, in the previous case both LAD and vein graft with a normal lumen at surgery, in 12 months became critically stenotic (90% and 80% respectively). Regional myocardial dysfunction is an important cofactor in accelerating atherosclerosis lesion in related artery.

## Target of ultrasound diagnosis: the present and the future

In the past years, clinical methods available to measure collateral flow have been too crude and showed major limitations, thus contributing to debate and confusion about the functional relevance of collateral circulation in the human myocardium. Coronary angiography allows visualization of collateral vessels having a diameter ≥100 μm, that actually prevents the majority of them from being detectable with this technique [16,17]. On the other hand, scintigraphic perfusion imaging techniques have limited spatial resolution [18]. Intracoronary wedge pressure and Doppler flow velocity measurements clearly demonstrated the presence of considerable collateral flow even in patients without angiographic evidence of collaterals [19,20], but they are invasive and not suitable for routine clinical use. With the introduction of new generation echo contrast agents and advanced ultrasound techniques, myocardial contrast echocardiography (MCE), an ultrasound imaging technique that utilizes physiologically inert gas-filled microbubbles as red blood cell tracers, has gained importance for the non-invasive assessment of blood flow at the level of myocardial perfusion [21,22]. Although evaluation of viability is the main clinical application of MCE [23], indirect assessment of collateral derived myocardial perfusion has been described in different clinical and experimental settings. In patients with severe left coronary artery disease, the placement of a graft to the posterior descending coronary artery was found to improve the collateral derived peak contrast effect within the anterior left ventricular wall [24]. In a series of subjects with healed myocardial infarction and total occlusion of the culprit vessel, a correlation was found between angiographic collateral grade and peak contrast effect after contralateral intracoronary contrast injection [25]. Collateral perfusion detected by MCE paralleled changes detected by radiolabeled microspheres during thrombosis and vasodilator administration in a canine model [26]. The usefulness of MCE has been confirmed in subjects without coronary occlusion where it was able to map the myocardial territory perfused by coronary collateral flow and to evidence immediate reduction of perfusion when collateral flow was abolished by angioplasty [27]. In patients with no prior myocardial infarction undergoing coronary angiography, intracoronary MCE effectively quantified coronary collateral flow, as demonstrated by the linear correlation existing between peak echo contrast effect and collateral flow index determined by intracoronary wedge pressure [28]. On the other hand, a strong correlation was reported between collateral receiving area at MCE and regional wall motion score index in patients with coronary occlusion, thus providing evidence that collateral derived perfusion is a good indicator of preserved regional function [29]. Likely, the grade of collateral flow on MCE was inversely correlated to the infarct size and was able to predict functional improvement following coronary revascularization [30]. Using an experimental model of chronic ischemia, it was found that not only the presence of collaterals can be identified by MCE, but also that temporal and spatial development of collateral circulation can be tracked serially [31].

Finally, intravenous MCE has been recently reported to provide qualitative and quantitative evaluation of collateral blood flow in the presence of an occluded infarct-related artery, and to emerge as the only predictor of true collateral blood flow among other markers [32].

All these reports as a whole support the concept that MCE provides important information on collateral flow and represents a promising mean for evaluating the status of coronary collateral circulation in clinical practice. Some important *caveat*, however, have to be taken into account. First, although the peak contrast pixel intensity has been reported as the most accurate of the variables obtained to measure collateral flow, there is a remarkable scatter in the correlation between peak pixel intensity and true collateral flow [33]. Second, it is known that regional contrast heterogeneity is common, resulting in frequent false positive perfusion defects [34]. Finally, coronary collateral vessels may cause additional dilution of contrast affecting the transit rate calculation. Further technical improvements may contribute in the near future to ensure standardization of the acoustic window and provide a quantitative evaluation of collateral flow. These issues appear to be of crucial importance to turn the echocardiographic assessment of coronary collateral flow into a ready-to-go clinical tool.

Besides the attempt to obtain direct echocardiographic assessment, coronary collateral circulation can indirectly affect the result of diagnostic stress testing with the use of echocardiographic technique. Increased vulnerability to myocardial ischemia induced by pharmacological coronary vasodilation was reported consistently with the hypothesis of a facilitated steal phenomenon in the presence of good collateral circulation [35]. On the other hand, the role of collaterals against echocardiographically-assessed stress-induced myocardial ischemia is controversial, some Authors reporting a protective [36] and others a neutral [37] effect. However, dobutamine-induced wall motion worsening in myocardial territories supplied by occluded epicardial vessels has been reported in case of evident collateral circulation [38], thus emphasizing the importance of a preserved, though reduced, blood flow to distinguish jeopardized myocardium from necrotic tissue. Differently, the ability of low-dose dobutamine stimulation to identify myocardial regions with a high probability of functional improvement after revascularization seems to be independent of both severity of underlying coronary stenosis and degree of collateralization of the involved coronary vessel [39].

The application of low-frequency ultrasound to intravascular microbubble contrast agents has been receiving attention in the last few years due to its potential therapeutic application, primarily as targeted gene delivery systems [40]. Further evidence from experimental studies has shown small capillary ruptures in exteriorized rat skeletal muscle [41], intact mouse muscle [42] and rabbit myocardium [43] to follow the application of ultrasound power. However, capillary rupturing via microbubble destruction with ultrasound is able to enhance arterioles per muscle fiber, arteriole diameters, and maximum nutrient blood flow in skeletal muscle [44]; thus, it may be tailored to stimulate an arteriogenesis response that restores hyperemia blood flow following arterial occlusion [45]. The potential of this method to become a clinical tool for stimulating blood flow to organs affected by occlusive vascular disease and, in particular, to the myocardium represents an interesting track for future research involving the application of ultrasound technology in the ischemic heart disease.

## Final consideration on coronary atherosclerotic plaque

Any hypothesis on the pathogenic role of a plaque and its activity and vulnerability should consider all interrelated variables for a correct interpretation of findings. When only one or few variables are investigated erroneous conclusions can be reached. An atherosclerotic plaque is always an active structure since its progression depends on a sequence of events due to a variety of correlated phenomena; while vulnerability is just an hypothesis which believe that some findings indicate a risk of plaque rupture.

The known variables are: degree of lumen reduction, shape, length, satellite collaterals, tunica media changes, inflammatory reaction per se and associated with media nerves (medial neuritis), survival (Table 3) macrophagic repair process, inflammation, vascularization hemorrhage, proteoglicans, atheroma, calcification, smooth muscle cell and elastic fiber hyperplasia, rupture, thrombosis, various factors released from all involved cells, hemodynamic pressure stresses, regional myocardial asynergy, spasm plus still unknown variables to be included.

**Table 3:** Occlusive coronary thrombus versus significantly main correlated variables. Percentage distribution

**Source**	**Acute myocardial infarct**	**Sudden unexpected death**
**Cases Total**	**200**	**208**
**Cases+occlusive thrombus%**	**41**	**15**

**Lumen reduction%**		
≤ 69	7	-
70–79	33	16
80–89	35	47
> 90	24	38
**Length stenosis mm**		
≤ 5	6	6
5–20	38	19
> 20	56	75
**Concentric**	100	94
**Atheromatous**	84	75
**Medial neuritis**	92	92
**Infarct size %**		
≤ 10	20	-
11–20	32	-
21–30	48	-
31–40	44	-
41–50	78	-
> 50	86	-
**Survival days**		
≤ 2	29	
3–10	51	
11–30	45	
**Survival minutes**		
< 10	-	12
10–60	-	23
61–180	-	30

Most studies analized few variables mainly observed in animals after hypercholesterol diet or in familial hypercholesterolemia. A pattern [46,47] totally different from that seen in general population and CHD. Furthermore, myocardial infarction is not synonymous of sudden/unexpected death, thrombus is a totally divergent structure from coagulum, collaterals can not be ignored and meaning of the coronary atherosclerotic plaque can be interpreted in another way.

**Figure 8:**
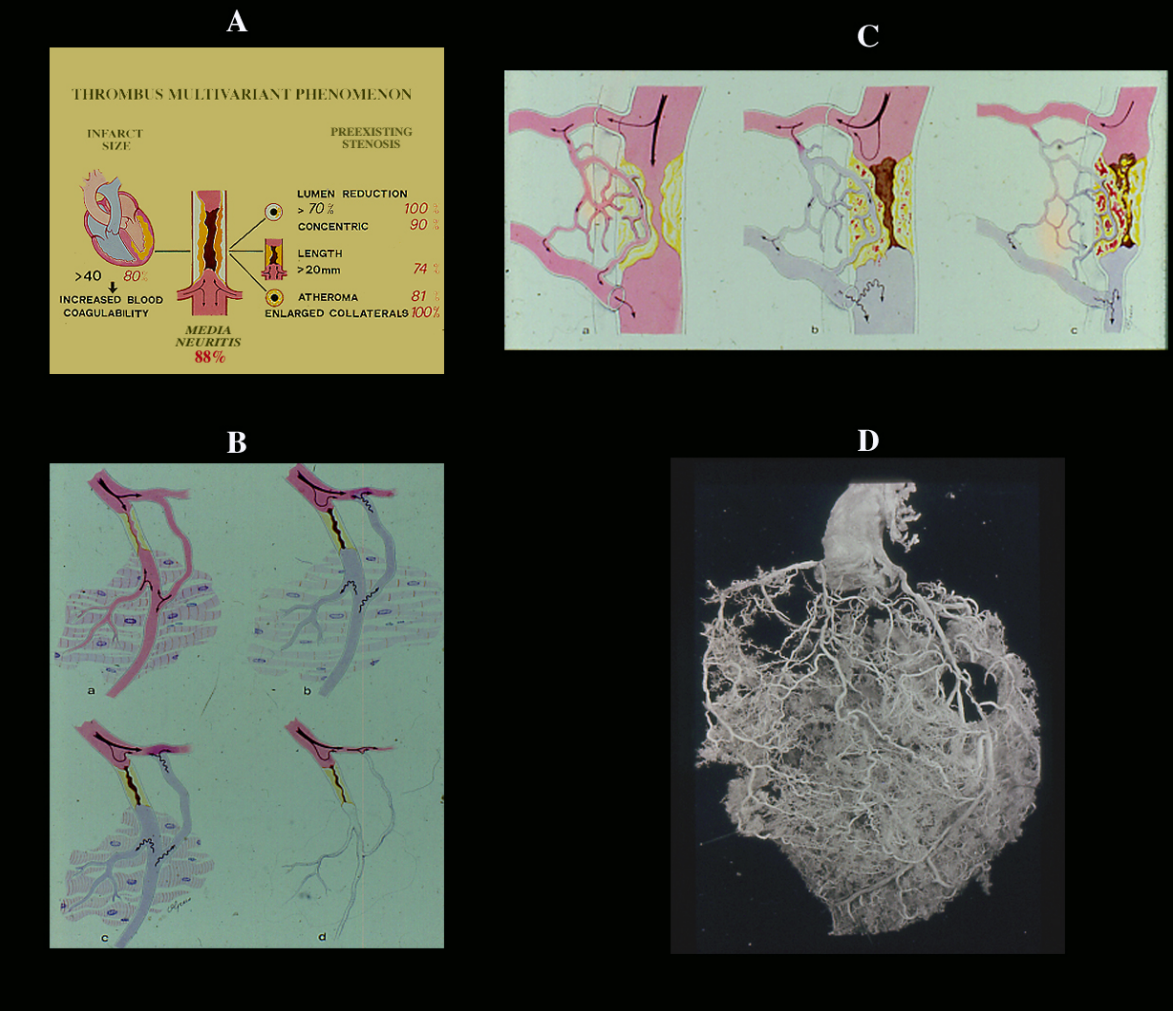
The coronary thrombus is a multivariant phenomenon (A), including medial neuritis. Its location in severe (≥70) stenosis associated with other factors (retrograde collateral flow, reduced fibrinolytic activity, etc, see text) justifies the concept that is a secondary phenomenon. Any time there is an increased peripheral resistance (B) (spasm, intramural extravascular compression following infarction, etc), stasis in related main vessel and in collaterals both outside and within the plaque is expected with hemorrhage, plaque rupture and trombosis (C). On the other hand, it is difficult to accept that acute occlusion of a pin-point lumen bypassed by preexisting functioning collaterals (D) may result in infarct necrosis or sudden death. Even experimentally occlusion of a severe "chronic" (7 days) stenosis does not produce any ischemic dysfunction.

The presence of functioning collaterals induces a particular hemodynamic condition within the residual lumen at the plaque level with proximal flow reduction counterbalanced by distal collateral flow. Any time there is a regional asynergy (Figure 8) with increasing intramural resistance, stasis in related artery will result in blockage of flow within the lumen with the most favourable situation for intimal hemorrhage, rupture, and thrombosis as secondary phenomena and not primary cause of an infarct. It is hard to believe that occlusion of a pinpoint lumen already compensated by collaterals is the cause of an infarct and rupture of a cap causes infarct or sudden death; being clear that any acute coronary syndrome is an etiopathogenetic entity which can not be caged in any unifying theory [48]. In the next review on different types of myocardial damage, this argument will be further reconsidered.

## Authors' contributions

Prof. Giorgio Baroldi contributed to the conception and organization of this review and to the final comments. Dr. Riccardo Bigi and Dr. Lauro Cortigiani summarized the use of ultrasound techniques in atherosclerotic plaque imaging.
